# Three Cases of Neoplastic Meningitis Initially Diagnosed with Infectious Meningitis in Emergency Department

**DOI:** 10.1155/2013/561475

**Published:** 2013-06-06

**Authors:** Shin Ahn, Kyung Soo Lim

**Affiliations:** Department of Emergency Medicine, Asan Medical Center, College of Medicine, University of Ulsan, 388-1, Pungnap-dong, Songpa gu, Seoul, 138-736, Republic of Korea

## Abstract

Neoplastic meningitis (NM) is diagnosed by the presence of malignant cells in the cerebrospinal fluid (CSF). We report 3 patients with NM, who were misdiagnosed with infectious meningitis in emergency department (ED). Case 1. A 68-year-old man visited our ED with a 3-month history of headache. With MRI and CSF study, he was diagnosed with tuberculous meningitis. After 20 days, repeated CSF cytology showed malignant cells. His diagnosis was lung cancer with NM. Case 2. A 57-year-old man visited regional hospital ED with a 3-week history of headache and diplopia. Brain MRI was not contributory. With CSF examination, his diagnosis was aseptic meningitis. With worsening headache, he was referred to our ED. Repeated CSF showed malignant cells. His diagnosis was stomach cancer with NM. Case 3. A 75-year-old man visited a regional hospital with headache lasting for 4 months. His diagnosis was sinusitis. Persistent symptom brought him back, and he developed recurrent generalized seizures. Brain MRI showed diffuse leptomeningeal enhancement suggesting meningitis, and he was transferred to our ED. CSF exam showed malignant cells. His diagnosis was NM with unknown primary focus. When evaluating the patients with headache in ED, NM should be kept in mind as a differential diagnosis of meningitis.

## 1. Introduction

Leptomeningeal carcinomatosis, so-called neoplastic meningitis (NM), is one of the complications of advanced cancer, occurring in 3–8% of all cancers with major neurological disability and high mortality [1]. Diagnosis of NM is made by clinical manifestations with appropriate findings on neuroimaging study or by examination of the cerebrospinal fluid (CSF) for the presence of malignant cells. However, the sensitivity of the initial CSF is approximately 50–60% and that of the enhanced magnetic resonance imaging (MRI) of brain is about 70% in diagnosing NM [2]. 

In this paper, we report 3 patients without previous history of malignancy, whose initial presentation was NM, but were misdiagnosed with meningitis of infectious origin in emergency department (ED) (Table 1).

## 2. Case 1

A 68-year-old man presented to our ED with a 3-month history of headache, which was managed at a private clinic but showed no improvements. His past medical history included hypertension for 10 years and pulmonary tuberculosis which was diagnosed 20 years ago but did not receive any treatment. His headache was continuous with dull ache in the frontal area, associated with nausea and low-grade fever. 

**Figure 1:**
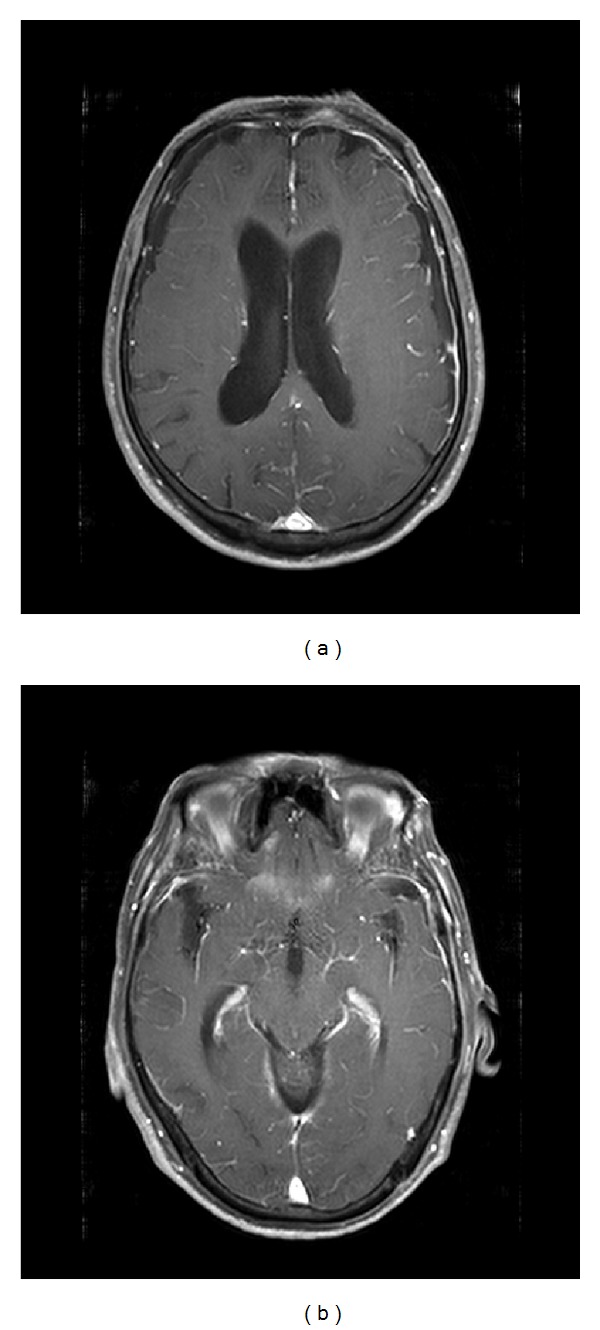
Brain magnetic resonance imaging with contrast showed leptomeningeal enhancement without parenchymal lesion.

On initial examination, he appeared well, in no acute distress, and had stable vital signs. He showed mild nuchal rigidity, but there were no neurological deficits. Initial hematology and chemistry panels and coagulation profile were all within normal limits. His precontrast brain computed tomography (CT) was unremarkable, and chest X-ray and CT scan of thorax showed patchy consolidation with ill-defined borders in right middle lobe, suspicious of pulmonary tuberculosis. Lumbar puncture was performed to rule out meningitis of tuberculous infection based on chest radiograph. CSF opening pressure was 15 cm H_2_O, with white blood cell (WBC) count of 110/mm^3^ (97% lymphocytes), 0/mm^3^ red blood cell (RBC), 419.3 mg/dL protein, 40 mg/dL glucose, and adenosine deaminase (ADA) of 12 U/L. His initial diagnosis was tuberculous meningitis, and he was admitted to neurology department and started antituberculous medications. CSF acid fast bacilli stain and culture were all negative, and polymerase chain reaction-hybridization for mycobacterium tuberculosis was also negative. However, seven days after the empirical treatment for tuberculosis, repeated CSF study showed improving CSF leukocytosis, and he was discharged for outpatient followup. Two weeks later, the patient revisited ED with persistent headache, nausea, and vomiting. Follow-up lumbar puncture was done showing WBC of 80/mm^3^ (85% lymphocytes), 0/mm^3^ RBC, 709.8 mg/dL protein, 50 mg/dL glucose, and ADA of 17 U/L. Cytospin of the CSF showed no evidence of malignancy. Contrast-enhanced brain MRI showed leptomeningeal enhancement without parenchymal lesion (Figure 1). Chemistry showed elevated aspartate aminotransferase (AST)/alanine aminotransferase (ALT) of 193/282 IU/L, which was assumed to be toxicity of anti-tuberculous medications and received conservative management. However, 4 days later, the CSF cytology smear showed malignant cells of metastatic carcinoma. Percutaneous needle aspiration of lung lesion which was initially suspected to be of tuberculosis was done and revealed well-differentiated adenocarcinoma. The patient was diagnosed with nonsmall cell lung cancer with leptomeningeal carcinomatosis; however, due to his old age, the patient did not receive any cancer treatments. He was expired 10 weeks later.

## 3. Case 2

A 57-year-old man visited our ED with a 3-week history of headache, vomiting, and diplopia. This patient visited an ED of a regional hospital 2 weeks ago, and after contrast-enhanced brain MRI which did not show any significant findings, CSF examination was done. According to the referred doctor, the CSF opening pressure was 33 cm H_2_O, with 26/mm^3^ WBC (50% lymphocytes), 0 RBC, 48 mg/dL protein, and 74 mg/dL glucose. His initial diagnosis was aseptic meningitis, and he received conservative treatment including mannitol loading for increased intracranial pressure. However, even after a week of treatment, the patient's symptoms did not improve and he was referred to our ED.

The patients' past medical history was unremarkable except for recently diagnosed noninsulin-dependent diabetes mellitus. He complained of weight loss of 10 kg within the last two months. Upon physical examination, the patient was alert but looked chronically ill. His extraocular muscle movement showed limitation of lateral gaze in his right eye, suggesting 6th cranial nerve palsy. Repeated lumbar puncture was done with an opening pressure of 38 cm H_2_O, with 8/mm^3^ WBC, 45/mm^3^ RBC, 50.2 mg/dL protein, and 64 mg/dL glucose. Differential count showed 80% of malignant cells suggesting leptomeningeal carcinomatosis. To find the primary cancer, chest and abdomen CT scan were done, showing negative results. Endoscopic exam performed on the next day showed stomach cancer with poorly differentiated adenocarcinoma. His final diagnosis was advanced gastric cancer with leptomeningeal carcinomatosis. He received three cycles of systemic chemotherapy. However, the patient died after 8 weeks of presentation due to persistent NM.

## 4. Case 3

**Figure 2:**
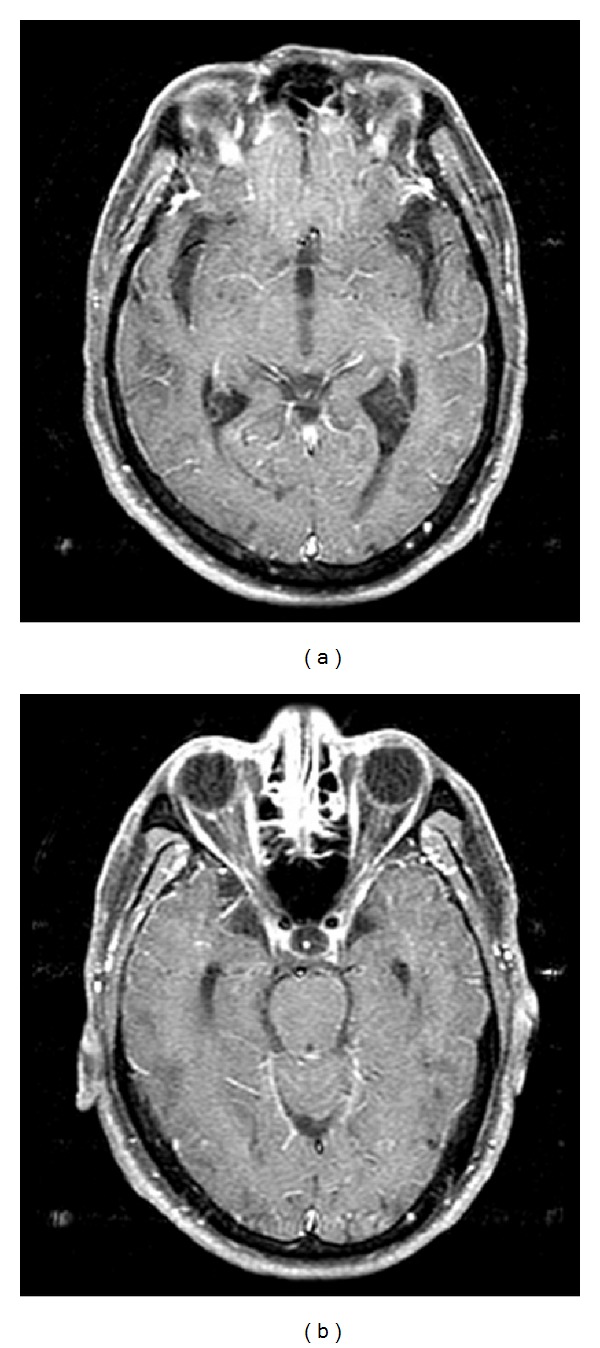
Brain magnetic resonance imaging with contrast showed mild diffuse ventriculomegaly and diffuse leptomeningeal enhancement, especially in posterior fossa area.

A 75-year-old man visited our ED with recurrent seizure attack during the management of chronic headache in a regional hospital. One week earlier, he visited an ED in a regional hospital with aggravating chronic headache lasting for 4 months. His brain CT was unremarkable except for sinusitis in the right maxillary sinus, and he was discharged with antibiotics and analgesics. However his symptoms did not improve, and he visited the same ED last night. During his stay in the ED, he developed generalized seizure lasting for 1 minute, and after the ictus, he became alert. Contrast-enhanced brain MRI was done, showing diffuse leptomeningeal enhancement in posterior fossa area, suggesting meningitis (Figure 2). He received antibiotics without CSF study. On the day of presentation, he had recurrent generalized seizure lasting for 1 minute with postictal confusion, and he was transferred to our ED.

On physical examination, he was afebrile and alert but confused and showed mild nuchal rigidity. His past history was unremarkable except for hyperlipidemia. CSF tapping was done, showing pressure of 9.5 cm H_2_O, WBC 120/mm^3^ (monocyte 87%), RBC 10/mm^3^, protein 94.6 mg/dL, and glucose 70 mg/dL. Suspecting meningitis associated with sinusitis, empirical broad spectrum antibiotics were given. His electroencephalography showed no epileptiform discharge. On the next day, CSF cytospin was reported showing 4% malignant cells. Chest and abdominal CT scan showed no primary cancer. His final diagnosis was leptomeningeal carcinomatosis with unknown primary focus. He received two cycles of intrathecal chemotherapy. However, due to old age and intolerance to chemotherapy, he stopped receiving cancer treatment, and the patient died of progression of neurological deterioration after 4 weeks.

## 5. Discussion

 Diagnosis of NM can be made by clinical findings, CSF cytology, and neuroimaging studies. NM could be suspected when patients with cancer show headache, meningeal irritation sign, various cranial nerve symptoms, and sometimes encephalopathy. However, these clinical signs and symptoms are often nonspecific and subtle, mimicking side effects of cancer chemotherapeutic agents, and benign condition could also produce these manifestations [3]. Moreover, in patient without known malignancies, NM could hardly be a differential diagnosis of priority. CSF cytology is estimated to have >95% specificity for NM [4]. However, the shedding of malignant cells could occur intermittently and in low volume, and its levels could vary in different levels of the neuraxis. Therefore, initial spinal fluid examination has low sensitivity ranging from 50 to 60%, which can be improved with a second CSF evaluation to approximately 80% [5]. Other CSF findings have limited sensitivity: increased opening pressure in 50–70%, elevated protein, and low glucose in 75% and 40%, respectively [6]. A gadolinium-enhanced MRI is more sensitive than CT scan for the detection of meningeal metastases, and at this time, enhanced MRI remains the procedure of choice [7]. However, this modality also has limited role with ≥30% incidence of false negative rate [8]. Due to these limitations among diagnostic modalities, diagnosing NM could be challenging, especially when the patient has no known history of malignancies.

Standard treatment of NM has traditionally involved radiotherapy to sites of symptomatic or bulky disease detected by neuroimaging, and in selected patients, administration of chemotherapy through intrathecal, intraventricular, or systemic route could be made. However, treatment remains palliative, and its prognosis is very poor, with reported median survival of 4–6 weeks when untreated, and 2–6 months with treatments [8]. 

The above reported cases were related to diagnosis of NM in patients without history of malignancies. These patients had in common relatively long duration of headache before presentation. Their brain MRI showed no parenchymal metastasis, whose diagnosis would not be a challenge when presented. CSF findings showed indistinctive features compared with infectious meningitis. In cases 1 and 2, detection of malignant cells was able only after the repeated CSF studies. All of the patients died soon because of the progressive neurological deterioration.

Repeated CSF study might not be a routine workup during the patients' stay in ED. However, when evaluating patients with persistent headache with relatively long duration, and considering its grave prognosis, NM should be kept in mind as a differential diagnosis of meningitis.

## Figures and Tables

**Table 1:** Summary of 3 cases of neoplastic meningitis.

Case	Complaints	Initial diagnosis	Initial CSF study	Positive malignant cell	MRI	Final diagnosis
68/M	Headache for 3 months	Tuberculous meningitis	Opening pressure 15 cm H_2_O WBC 110/mm^3^ (97% lymphocytes) RBC 0/mm^3^ Protein 419.3 mg/dL Glucose 4 mg/dL Adenosine deaminase 12 U/L Cytospin: no malignant cell	3rd CSF	Leptomeningeal enhancement without parenchymal lesion	Nonsmall cell lung cancer with leptomeningeal carcinomatosis
57/M	Headache for 3 weeks, diplopia	Aseptic meningitis	Opening pressure 33 cm H_2_O WBC 26/mm^3^ (50% lymphocytes) RBC 0/mm^3^ Protein 48 mg/dL Glucose 74 mg/dL Cytospin: no malignant cell	2nd CSF	No abnormal finding	Advanced gastric cancer with leptomeningeal carcinomatosis
75/M	Headache for 4 months, recurrent seizure	Unspecified meningitis	Opening pressure 9.5 cm H_2_O WBC 120/mm^3^ (87% monocyte) RBC 10/mm^3^ Protein 94.6 mg/dL Glucose 70 mg/dL Cytospin: 4% malignant cell	1st CSF	Leptomeningeal enhancement in posterior fossa without parenchymal lesion	Leptomeningeal carcinomatosis with unknown primary focus

CSF: cerebrospinal fluid; MRI: magnetic resonance imaging; WBC: white blood cell; RBC: red blood cell.
